# Comparison of malaria diagnostic methods for detection of asymptomatic *Plasmodium* infections among pregnant women in northwest Ethiopia

**DOI:** 10.1186/s12879-024-09369-y

**Published:** 2024-05-14

**Authors:** Adane Tilahun, Mulat Yimer, Woynshet Gelaye, Banchamlak Tegegne, Demeke Endalamaw, Fikirte Estifanos, Abtie Abebaw, Aberham Abere

**Affiliations:** 1 Department of Medical Laboratory Science, College of Medicine and Health sciences, Debre Markos University, Debre Markos, Ethiopia; 2 Department of Medical Laboratory Sciences, School of Health Sciences, College of Medicine and Health Sciences, Bahir Dar University, Bahir Dar, Ethiopia; 3 Amhara Public Health Institute, Bahir Dar, Ethiopia; 4 School of Biomedical and Laboratory Sciences, College of Medicine and Health sciences, University of Gondar, Gondar, Ethiopia

**Keywords:** Asymptomatic infection, *Plasmodium*, Pregnant women, Jawi, Ethiopia, Diagnostics, PCR

## Abstract

**Background:**

Malaria in pregnancy remains a major public health problem in the globe, especially in sub-Saharan Africa. In malaria endemic areas, most pregnant women remain asymptomatic, but malaria could still cause complications on the mother and her offspring; as well as serve as reservoirs to transmit infection. Despite these effects, no attention is given to the diagnosis of asymptomatic *Plasmodium* infections (APIs) using highly sensitive and specific laboratory diagnostic tools in Ethiopia. Therefore, the goal of this study was to compare the performance of Rapid Diagnostic Test (RDT), microscopy and real-time polymerase chain reaction (RT-PCR) to detect APIs among pregnant women.

**Methods:**

A health facility based cross -sectional study was conducted among pregnant women attending antenatal care at Fendeka town health facilities Jawi district, northwest Ethiopia from February to March, 2019. A total of 166 participants were enrolled by using convenient sampling technique. Socio-demographic features were collected using a semi structured questionnaire. Dried blood spot (DBS) samples were collected for molecular analysis. Asymptomatic *Plasmodium* infection on pregnant women was diagnosed using RDT, microscopy and RT-PCR. Descriptive statistics were used to determine the prevalence of APIs. Method comparison was performed, and Cohen’s kappa coefficient (k) was used to determine the degree of agreement among the diagnostic methods. Parasite densities were also calculated.

**Results:**

The prevalence of API was 9.6%, 11.4% and 18.7% using RDT, microscopy and RT-PCR, respectively. The overall proportion of API was 19.3%. Sensitivity of the RDT was 83.3% as compared with microscopy. Rapid Diagnostic Test and microscopy also showed sensitivity of 50% and 60%, respectively, as compared with RT-PCR. The mean parasite density was 3213 parasites/µl for *P falciparum* and 1140 parasites/µl of blood for *P. vivax*.

**Conclusion:**

Prevalence of API in the study area was high. Both RDT and microscopy had lower sensitivity when compared with RT-PCR. Therefore, routine laboratory diagnosis of API among pregnant women should be given attention and done with better sensitive and specific laboratory diagnostic tools.

## Background

*Plasmodium* species cause the protozoan disease known as malaria, which is spread through the bite of infected female Anopheles mosquitoes [1]. It still poses a serious health problem, especially to pregnant women, the developing foetus, and the infant in tropical and subtropical areas of the world. Twenty-five million pregnant women are currently at risk for malaria, and, according to the World Health Organization (WHO), malaria accounts for over 10,000 maternal and 200,000 neonatal deaths per year [2]. In the WHO African Region, there were an estimated 13.3 million malaria cases during pregnancy in 2021 [1]. In Ethiopia, according to PMI report from 2008 to 2009, pregnant women accounted for 1.7% out of all reported outpatients with malaria, 2.9% of malaria hospitalizations, and 1.7% of inpatient malaria deaths [3]. Compared to other species, *Plasmodium falciparum* is mostly responsible for complications that happen during pregnancy [4]. Maternal anaemia is the most frequent and potentially fatal complication of pregnancy related malaria [5].

People are frequently exposed to *Plasmodium* species in malaria endemic areas, which promotes the development of partial immunity. This immunity protects against potentially fatal parasite burdens and suppresses the pro-inflammatory reactions that cause illness, making infected people, including pregnant women, asymptomatic. Because asymptomatic people do not seek treatment, act as reservoirs, and propagate the disease. APIs are also the main barrier to malaria control and elimination programmes [6, 7].

Asymptomatic *Plasmodium* infections produce a placental infection during pregnancy [8, 9] which results in a localized inflammatory response and a significant infiltration of immune cells such as macrophages and lymphocytes [10, 11]. This condition disrupts the mother-to-fetus flow of nutrients and oxygen, which raises the risk of foetal mortality, preterm, low birth weight, abortion, stillbirths, and foetal anaemia [12, 13].

Asymptomatic infections do not have sign and symptoms of malaria and even do not detect by less sensitive routine diagnostic tools due to low parasite densities. In many places where malaria is endemic, microscopic examination is still the gold standard for diagnosing the disease. Although microscopy can be used to distinguish between different parasite species and provide quantitative data on parasite densities, it has significant drawbacks, including limited sensitivity [14, 15], which calls for better diagnostic techniques for malaria elimination programmes. Rapid diagnostic tests have become crucial alternative tool for malaria diagnosis even if, some RDTs are *Plasmodium* specific, detecting the genus-specific aldolase and the recent discovery that some *P. falciparum* parasites in parts of South America and Africa have deleted the hrp-2 gene [16, 17] has raised a question on the use of the HRP-2 based RDTs due to false negatives. In addition, the specificity of the commonly used RDT that detects histidine rich protein–II (HRP-II) of *P. falciparum* is limited when the parasite is cleared, and antigens remain in circulation for about 28 days [18].

Another drawback of RDTs is that they cannot be used to determine parasite densities, even though such quantification is essential for both antimalarial drug resistance surveillance and malaria control programs [19]. Due to the above mentioned reasons, comparing the results of both RDT and microscope with highly sensitive and specific methods i.e. .RT-PCR specifically for the diagnosis of APIs is very important to scaling up the success of malaria control and elimination programs [20]. So, our main objective was to assess the performance of RDTs with microscopy, performance of RDT and microscopy with PCR for detection of asymptomatic *Plasmodium* infections, prevalence, and parasite densities of APIs among pregnant women.

The three major vector control measures, namely environmental management, use of indoor residual spraying (IRS) and insecticide treated bed nets (ITNs) have been implemented in Ethiopia [21]. Surveillance and early diagnosis and prompt treatment are also the key control strategies [2].

## Methods and materials

### Study design, period and area

**Fig. 1:**
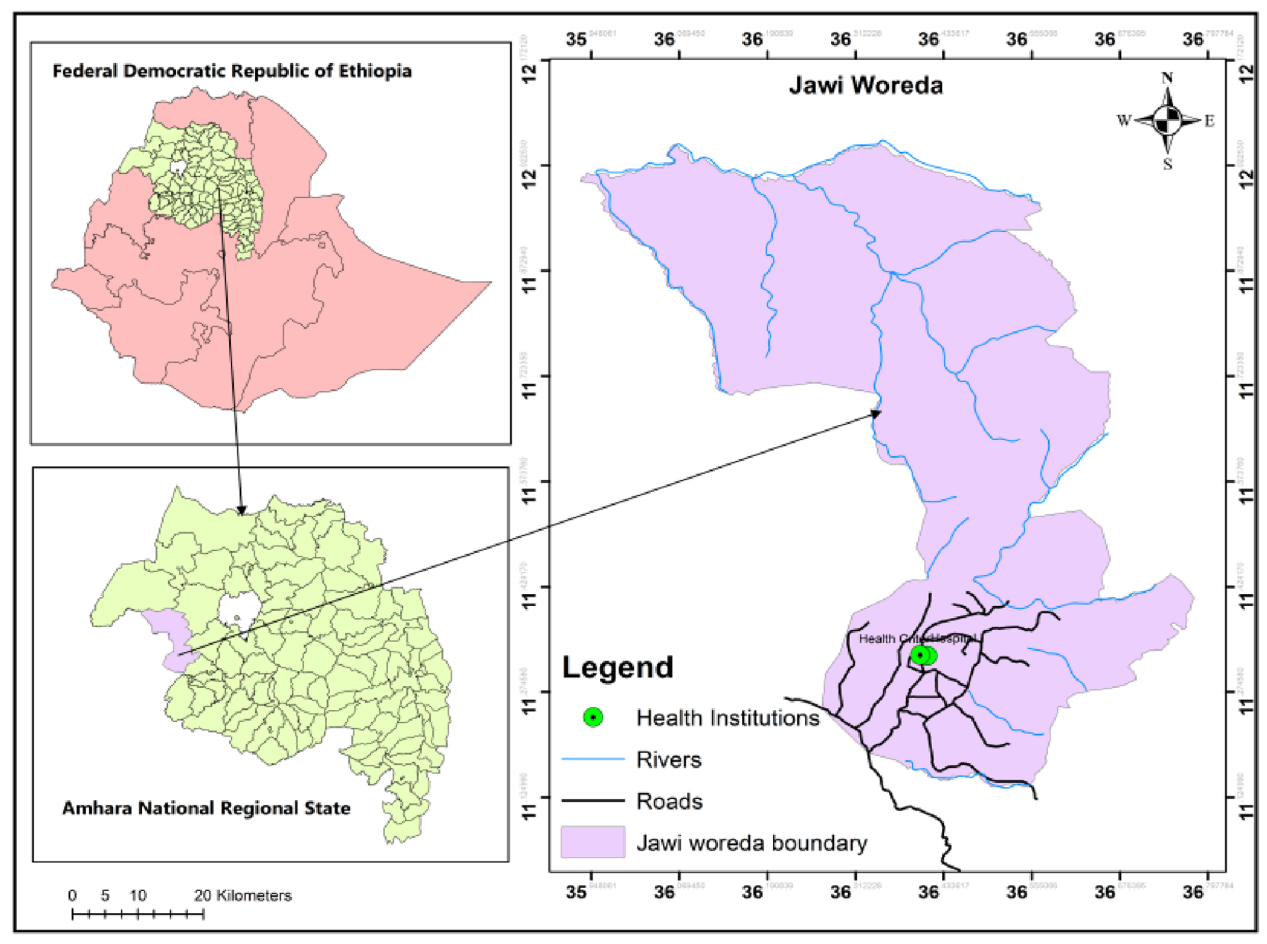
Map of the Federal Democratic Republic of Ethiopia and Amhara Regional State showing the study area, Jawi district

A health facility based cross-sectional study was conducted from February 1st – March 30th, 2019, which is outside of the main malaria transmission season at Fendeka town Jawi district, Northwest Ethiopia (Fig. 1). Jawi district is one of the districts with endemic malaria transmission in the Amhara region of Ethiopia. The study was done within this district in Jawi general hospital and Jawi health center. The mean temperature varies between 16.68^0^c and 37.6^0^c and the altitude ranges from 648 to 1300 m above sea level. The number of malarias reported cases of the district in 2018/2019 was 24,434 (Jawi district health office malaria case report). Additionally, the research area is home to the Tana Beles integrated sugar production project and malaria transmission occurs all year round. The district had a total population of 79,090 people as of the 2007 National Central Statistical Census Report, of which 41,407 were men and 37,683 were women [22]. This area also has long summer rain fall (June-September) and a winter dry season (October-May) with mean annual rain fall of 1569.4 mm [23].

### Sample size determination and sampling techniques

Sample size was calculated by Buderer’s formula using 39% prevalence 56.4% sensitivity, 90% specificity of RDT [24]; and 12% error rate. A total of 166 pregnant women were participated in the study. An equal number of 83 participants were selected from both Jawi General Hospital and Jawi Health Center using convenient sampling technique.

### Data collection materials methods

#### Socio-demographic characteristics

Midwives gave identification card to pregnant women and examined the status of their pregnancy during their antenatal care follow up. After checking their status and if they were free from any malaria related sign and symptoms, they were recruited and requested their willingness to participate in the study. From volunteered pregnant women, socio-demographic characteristics and previous use of anti-malarial drugs were collected via semi structured questionnaire by the trained midwives.

### Inclusion and exclusion criteria

Inclusion criteria: - Pregnant women who did not show sign and symptoms of malaria.

Exclusion criteria were: - Pregnant women who had taken anti-malaria drugs within the last four weeks, pregnant women whose axillary temperature was > 37.5°c and pregnant women who were chronically ill due to complicated pregnancy or with other illnesses.

Blood sample collection: Blood sample was collected by pricking at their ring finger for RDT, thick and thin blood films and DBS using Whatman filter paper. For RDT, 5 µl of capillary blood were added using micropipette to the sample well and 2 drops (60 µl) of buffer solution to the buffer well and then after waiting for 20 min, the results were reported [manufacturers leaflet] according to the manufacturer’s instruction. Thin and thick blood films were prepared by dropping 2–3 µl and 6–10 µl of blood sample respectively on a single slide and spreading on it [25]. Thick and thin films were air dried, and thin films were fixed by dipping in absolute methanol for 10–20 s. Then, slides were stained with 3% Giemsa for 30–45 min [26]. For DBS, 1–2 large drops of blood were spotted on Whatman903 filter paper (DBS card) which is made by Lasec Company in South Africa and allow them to air dry for 2–3 h. After the DBS cards were dried, they were packaged into each sealed plastic bags with desiccant; and it was stored at − 20 ^0^c in the lab. Later, it was transferred to Amhara Public Health Institute for molecular analysis using RT-PCR.

#### Laboratory diagnosis

Parasite detection by CareStart™ Malaria Pf / Pv (HRP2/pLDH) Ag Combo RDT was done based on manufacturer instructions. Thin and thick stained smears were examined under light microscope (Olympus CX-21) with 100x magnification for detection and identification of *Plasmodium* species by experienced laboratory professionals working at Jaw health centre and hospital. Thick blood films were considered positive when sexual and asexual forms were detected and considered as negative after observing 200 high power fields without detecting any parasite. *Plasmodium* parasites were counted against 200 leukocytes and expressed as number of parasites per microliter (µl) of blood (parasite density) with the assumption that there would be average white blood cell counts of 8000 /µl of blood [27].

Number of parasites/µl of blood = 8000*No. of asexual parasites counted against 200 WBCs/ 200. Parasitaemia was classified as low (< 500 parasite/µl of blood), moderate (501–5000 parasites/µl of blood) and high (≥ 5000 parasites/µl of blood) as described by Allen and colleagues [28].

For molecular analysis, we punched out single 3-mm punches of DBS, placed in to1.5-ml micro-centrifuge tubes, added 180 µl ATL buffer and incubated at 85 °C for 10 min. After incubation, the sample centrifuged briefly to remove drops from inside the lid and 20 µl proteinase K stock solution added, mixed by vortexing and incubated at 56 °C for 1 h. Then the sample was centrifuged; 200 µl AL buffer added and mixed immediately and thoroughly by vortexing and incubated at 70 ^0^C for 10 min. Again, the sample was centrifuged; 200 µl ethanol (96%) added and mixed by vortexing and then centrifuged. Then the mixture applied to the QIAamp Mini spin column, the cap closed, and centrifuged at 8000 rpm for 1 min. After centrifugation, the QIAamp Mini spin column placed in a clean 2 ml collection tube and discarded the tube containing the filtrate. 500 µl AW1Buffer added to the QIAamp Mini spin column, the cap closed and centrifuged at 8000 rpm for 1 min and placed in another clean 2 ml collection tube. Then 500 µl AW2 Buffer added to the QIAamp Mini spin column, the cap closed and centrifuged at 14,000 rpm for 3 min and placed in another clean 1.5 ml micro-centrifuge tube and again centrifuged at 14,000 rpm for 1 min. Finally, the QIAamp Mini spin column placed in another clean 1.5 ml micro-centrifuge tube, added 100 µl AE Buffer incubated at room temperature for 3 min, and then centrifuged at 8000 rpm for 1 min. The DNA was eluted in 100 µl of elution buffer, aliquoted and stored at -20 ^0^C until running PCR assay [29].

The PCR assay was run using Agilent Technologies strata gene Mx3005p PCR machine.

#### PET-PCR primer sequence

The photo-induced electron transfer (PET) primer consists of a quasi-universal tail sequence at 5’ end sequence specific region at 3’ (Table 1).

**Table 1:** Photo -induced electron transfer PCR (PET-PCR) Primer sequences

PET-PCR Primer types	Sequences
Original Genus 18sFor	5’-GGC CTA ACA TGG CTA TGA CG-3’
Original Genus FAM 18sRev	5’-agg cgc ata gcg cct ggC TGC CTT CCT TAG ATG TGG TAG CT-3’
Falciparum For	5’-ACC CCT CGC CTG GTG TTT TT-3’
Falciparum Rev, HEX	5’-agg cgg ata ccg cct ggT CGG GCC CCA AAA ATA GGA A-3’
P. vivax For	5’-GTA GCC TAA GAA GGC CGT GT-3’
P. vivax Rev, HEX	5’- agg cgc ata gcg cct ggC CTG GGG GAT GAA TAT CTC TAC AGC ACT GT-3’

For: Forward Rev: Reverse FAM AND HEX: Modified and lebeled with fluorescence dye 5’ end

Amplification of *Plasmodium* Genus and *P. falciparum* or *P. vivax* was performed in a 20 µl reaction containing 2X TaqMan Environmental Master Mix buffer, forward and reverse primers for the Genus and *P. falciparum* multiplex assays and *P. vivax* singleplex assay, and 5 µl of DNA samples. Samples were run in duplicate on manually loaded 96-well PCR plates. The reactions were performed under the following cycling conditions: initial hot-start at 95 ^0^C for 15 min, followed by 45 cycles of denaturation at 95 ^0^C for 20 s and annealing at 60 ^0^C for 40 s for Genus and *P. falciparum* multiplex assays.

For *P. vivax* single plex assay, initial hot-start at 95 ^0^C for 15 min, followed by 45 cycles of denaturation at 95 ^0^C for 20 s and annealing at 60 ^0^C for 40 s and then at 72 ^0^C for 30 s. The correct fluorescence channel was selected for each fluorescently labeled primer set and the cycle threshold (CT) values recorded at the end of annealing step. Any sample with a CT value of 40.5 or below was considered positive [24]. The Genus and *P. falciparum* multiplex assays were performed for all samples. *P. vivax* singleplex assay was performed for Genus positive and *P. falciparum* negative samples.

### Data quality control and management

The principal investigator (PI) trained the data collectors on how to gather the sample and complete the questionnaires prior to the data collection. Socio-demographic characteristics were collected by midwives. While skilled laboratory professionals were also involved for RDT, thin and thick blood film preparation, DBS sample collection, and *Plasmodium* species detection and identification by using, similar microscope Olympus CX-21 to minimize the possible instrumental error. The questionnaires were written in a clear and understandable manner, and the questions’ flow and clarity were carefully evaluated. Data were gathered under the close observation of the principal investigator (PI). Laboratory professionals who performed microscopy were blinded to RDTs results and before concluding the microscope result as negative, 200 fields of the thick smear were examined. Moreover, for microscopy reading both negative and positive control slides were used. In addition, for microscopy reading both negative and positive control slides were used. Similarly, for real-time PCR analysis negative and positive control samples were used in each run. Finally, all smeared slides were brought to Bahir Dar and rechecked by the investigator. Discordant results again rechecked with medical parasitologist in Amhara public health institute.

### Ethical approval and consent to participate

The study was approved by the ethics committee of college of medicine and health sciences, Bahir Dar University (with protocol number 0193/18 − 09, day 07 January 2019). Additionally, authorization letters were acquired from the managers of health facilities, the Amhara Public Health Institute, the Zonal Health Department, and the District Health Office. Written informed consent was obtained from the participants. Illiterate participants agreed to participate by giving their fingerprint signature after listening the written informed consent. All methods were carried out in accordance with relevant guidelines and regulations. Each participant’s information was only used for this study, and confidentiality was always upheld. Finally, the attending midwives obtained positive results and prescribe anti-malarial medication.

### Data analysis

Data were entered into Epi-data version 3.1, coded, cleaned then exported and analyzed using the statistical software SPSS version 20. Descriptive statistics were used to generate the general characteristics of the pregnant women, the prevalence of APIs among them and among different variables such as residence, age, gravidity and gestational age proportion of detected *Plasmodium* species among pregnant women, comparing performance of the three diagnostic methods based on sensitivity, specificity and predictive values within 95% confidence interval, the level of measure of agreement (k) between them and calculating mean parasite density.

## Results

### General characteristics of pregnant women

A total of 166 pregnant women were enrolled and, of these 124 (74.7%) were rural dwellers. Their age range was from 17 to 40 years and the mean age was 26 years. Many of the participants 46 (27.7%) were between 26 and 30 years of age. Majority 124 (74.7%) of study participants unable to read and write and farmers accounted for 136 (81.9%). The majority of 158 (95.2%) were married. Among the participants, 86 (51.8%) were multigravidae and 76 (45.8%) were at their third trimester (Table 2).

**Table 2:** General characteristics of the pregnant women attending antenatal care at Fendeka town health facilities from February - March 2019, (*N* = 166)

Variables.		*N* (%)
Residence	Rural	124(74.7)
	Urban	42(25.3)
Age Group	≤ 20	42(25.3)
	21–25	40(24.1)
	26–30	46(27.7)
	≥ 31	38(22.9)
Education level	Unable to read and write	124(74.7)
	Able to read and write but no formal education	2(1.2)
	Grades 1–6	6(3.6)
	Grades 7–10	20(12.1)
	Grades 11–12	10(6)
	Diploma and above	4(2.4)
Occupation	Farmer	136(82)
	GVT employee	4(2.4)
	Student	2(1.2)
	Housewife	14(8.4)
	Self/private employee	10(6)
Marital status	Married	158(95.2)
	Single	4(2.4)
	Divorced	2(1.2)
	Widowed	2(1.2)
Gravidity	Primigravidae	50(30.1)
	Secugravidae	30(18.1)
	Multigravidae	86(51.8)
Gestational age	1st trimester	26(15.7)
	2nd trimester	64(38.6)
	3rd trimester	76(45.8)

GVT: Government N: Number of participants

### Prevalence of asymptomatic *Plasmodium* infections

The prevalence of APIs was 16 (9.6%), 19 (11.4%) and 31 (18.7%) by RDT, microscopy and RT-PCR, respectively. The overall prevalence was 19.3% (Table 3).

**Table 3:** Prevalence of *Plasmodium* species among pregnant women attending antenatal care at Fendeka town health facilities from February – March 2019, (*N* = 166)

Plasmodium Species	Diagnostic Methods		
	RDTs *N* (%)	Microscopy *N* (%)	PCR *n* (%)
Pf	10 (6)	11 (6.6)	17 (10.2)
Pv	4 (2.4)	4 (2,4)	10 (6)
Mixed	2 (1.2)	4 (2.4)	4 (2.4)
Total N (%)	16(9.6)	19(11.4)	31(18.7)

Pf; Plasmodium falciparum Pv; Plasmodium vivax Mixed; Pf and Pv

A total of 32 asymptomatic malaria cases were detected by all the three diagnostic methods.

Out of 32 total malaria cases, 16 were detected by RDT, 19 detected by microscopy and 31 detected by RT-PCR. One *plasmodium falciparum* case detected by RDT was not identified by either microscopy or RT-PCR. Microscopy identified 2 more *plasmodium falciparum* and 2 mixed cases than RDT. Real time PCR also detected 16 and 12 more cases than RDT and microscopy respectively. The overall prevalence was 19.3% (Table 4).

**Table 4:** proportion of *Plasmodium* species among pregnant women attending antenatal care at Fendeka town health facilities from February – March 2019, (*N* = 166)

Plasmodium species	No. of plasmodium species detected	Percentage
Pf	18	10.8%
Pv	10	6%
Mixed	4	2.4%
Total	32	19.3%

Pf: Plasmodium falciparum Pv: Plasmodium vivax Mixed: Pf and Pv

The prevalence of asymptomatic malaria cases was higher in rural dwellers than urban, in *≤* 20 years of age than other age groups and in primigravidae than secugravidae and multigravidae. Asymptomatic plasmodium infection prevalence showed statistical significance association with age group and gravidity but did not show statistical significance association with residence and gestational age using chi square test (Table 5).

**Table 5:** prevalence of asymptomatic malaria cases on different variables among pregnant women attending antenatal care at Fendeka town health facilities from February – March 2019, (*N* = 166)

Variables		No. of plasmodium species detected			Total N (%)	Chi square test (P-value)
		Pf	Pv	Mixed		
Residence	Rural	14	10	4	28 (16.9)	0.07
	Urban	4	0	0	4 (2.4)	
Age	< 20	6	8	2	16 (9.6)	0.002
	21–25	2	0	0	2 (1.2)	
	26–30	6	0	2	8 (4.8)	
	≥ 31	4	2	0	6 (3.6)	
Gravidity	Primigravidae	6	6	4	16 (9.6)	0.01
	Secugravidae	4	2	0	6 (3.6)	
	Multigravidae	8	2	0	10 (6)	
Gestational age	1st trimester	0	2	0	2(1.2)	0,20
	2nd trimester	6	4	2	12(7.2)	
	3rd trimester	12	4	1	18(10.8)	

Many asymptomatic malaria cases were detected by all the three diagnostic methods and especially, those positive cases by PT-PCR but negative by both RDT and microscopy had high CT values. Relatively positive samples by all the three diagnostic methods and with high parasite densities of microscopically detected samples had low CT values. Most CT values were concordance with the parasite densities of positive tests detected by microscopy.

### Comparison of RDT with microscopy for detection of APIs

The diagnostic performance of RDT was assessed by taking thick blood film as a reference method. Among 19 microscopy confirmed infections, 15 of them were detected by RDT. The sensitivity, specificity, positive predictive value and negative predictive value of the RDT were 83.3%, 99.3%, 93.75%, and 98%, respectively. An excellent measure of agreement (k = 0.803) was obtained between the RDT and microscopy (Table 6).

**Table 6:** Comparison of RDT with microscopy to detect asymptomatic *Plasmodium* infections among pregnant women attending antenatal care at Fendeka town health facilities from February - March 2019, (*N* = 166)

Diagnostic Methods		Microscopy			Sensitivity (95% Cl)	Specificity (95% Cl)	PPV (95% Cl)	NPV (95% Cl)	K
		Pos	Neg	Total					
RDT	Pos	15	1	16	83.3(76.7–88.1)	99.3(96.7–99.9)	93.75(89.3–96.7)	98(94.8–99.4)	0.893
	Neg	3	147	150					
	Total	18	148	166					

### Comparison of RDT and microscopy verses PCR for detection of APIs

Rapid diagnostic test and microscopy performance were evaluated using RT-PCR as a reference technique. Out of 31 PCR confirmed infections, RDT detected only 15 and microscopy also detected 19. There was one positive result detected by RDT, but it was negative by both microscopy and PCR. The sensitivity, specificity, positive predictive values and negative predictive values of RDT were 50%, 99.3%, 93.75%, and 90%, respectively. The sensitivity, specificity, positive predictive value, and negative predictive value of microscopy were 60%, 100%, 100%, and 91.9%, respectively. A good measure of agreement (k = 0.62 and k = 0.71) was observed for RDT and microscopy with RT-PCR, respectively (Table 7).

**Table 7:** Comparison of RDT and microscopy with PCR to detect asymptomatic *Plasmodium* infections among pregnant women attending antenatal care at Fendeka town health facilities from February - March 2019, (*N* = 166)

Diagnostic Methods		RT-PCR			Sensitivity (95% Cl)	Specificity (95% Cl)	PPV (95% Cl)	NPV (95% Cl)	K
		Pos	Neg	Total					
RDT	Pos	15	1	16	50(42.3–57.5)	99.3(94.8–99.4)	93.75(89.3–96.7)	90(84.2–93.5)	0.62
	Neg	15	135	150					
	Total	30	136	166					
Microscopy	Pos	19	0	18	60(49.5–70.5)	100(95.6–100)	100(95.6–100)	91.9(86-97.8)	0.71
	Neg	11	136	148					
	Total	30	136	166					

PPV: Positive predictive value NPV: Negative predictive value K: Kappa value

### Parasite densities

Of the 19 asymptomatic infections detected by microscopy, (52.6%), (31.6%) and (15.8%) have low, moderate, and high parasite densities, respectively. For *P. vivax*, there were (75%) low and (25%) moderate parasite densities. Parasite densities ranged from 360 to 5120 parasites/µl of blood. The mean parasite density was 3213 parasites/µl for *P falciparum* and 1140 parasites/µl of blood for *P. vivax*. Parasite densities were concordance with the CT values of their PCR test results (Table 8).

**Table 8:** *Plasmodium* parasite densities among pregnant women attending antenatal care at Fendeka town health facilities from February - March 2019, (*N* = 19)

Plasmodium species detected by microscopy	Parasite density			Total
	Low ≤500	Moderate 501–5000	High > 5000	
*P. falciparum*	6	4	2	11
*P.vivax*	3	1	0	4
Mixed	1	2	1	4
Total N (%)	10 (52.6)	6 (31.6%)	3 (15.8%)	19

High parasite density (45%) was identified in *≤* 20 years of age than other age groups. The parasite density was 45.5% moderate and 36.4% high in primigravidae and 66.7% moderate in secugravidae (Table 9).

**Table 9:** Distribution of parasite densities in age groups and number of pregnancies among pregnant women attending antenatal care at Fendeka town health facilities from February – March 2019, *N* = 19

Variables		Parasite density			Total
		Low (< 500 parasites/µl)	Moderate (501–5000 parasites/µl)	High (> 5001 parasites/µl)	
Residence	Rural	6 (37.5%)	6 (37.5%)	4 (25%)	16 (84.2%)
	Urban	1 (33.3%)	2 (66.7%)	0	3 (15.8%)
Age	< 20	2 (18.2%)	4 (36.4%)	5 (45%)	11 (57.9%)
	21–25	2 (100%)	0	0	2 (10.5%)
	26–30	0	4 (100%)	0	4 (21.1%)
	≥ 31	2 (100%)	0	0	2 (10.5%)
Gravidity	Primigravidae	2 (18.2)	5 (45.5%)	4 (36.4%)	11 (57.9)
	Secugravidae	2 (33.3%)	4 (66.7%)	0	6 (31.6%)
	Multigravidae	2 (100%)	0	0	2 (10.5%)

## Discussion

Asymptomatic *Plasmodium* infections cause various major risks to pregnant women, the growing foetus, and neonates. It also could be an obstacle in malaria elimination programs due to low treatment seeking behaviors of infected individuals and absence of standardized diagnostic tools. As a result, they serve as reservoirs for sustainable malaria transmission [30].

In our study RT-PCR detected the highest proportion of APIs followed by microscopy and RDT. These differences might be due to variation in the detection limits; where PCR detects as low as 0.1 parasites **/** µl of blood [31]; while microscopy and RDTs detect 50 and 100–200 parasites/µl of blood, respectively [32].

One *P. falciparum* positive case in this investigation, out of 16 APIs detected by RDT, tested negative by RT-PCR and microscopy. This can be the result of transcriptional error while reporting, use of antimalarials in the past few days which eliminates parasites but HRP2 persists in the circulation or cross reactivity with other protozoan parasite antigens [33]. Out of the 19 APIs detected by microscopy, 15 were found by RDT and all 19 were found by RT-PCR. Rapid diagnostic test identified only 15 of the 31 PCR-confirmed illnesses, and microscopy also found 19 of them. When compared to RT-PCR, RDT and microscopy showed less sensitivity 50% and 60% and specificity 99.3% and 100%, respectively. This could be because of the RDT’s 98% sensitivity for diagnosing symptomatic cases [26] and the lower parasite densities in asymptomatic infections. However, there was good degree of agreement between the three laboratory diagnostic techniques. Out of 19 APIs detected by microscopies 44.4%, 38.9%, and 16.7% were low, moderate and high parasite densities, respectively. The mean parasite density was 3212 parasites/µl for *P. falciparum* and 1140 parasites /µl for *P. vivax.* Parasite densities were concordance with the CT values of their PCR test results. Percentage of moderate parasite densities was lower than the study done in south Ethiopia 74.2% [34]. The mean parasite density was also lower than the study done in Burkina Faso 4085 parasites/µl [10]. These differences might be due to variations in partial immunity within the community and malaria transmission intensity.

For *P. falciparum*, the parasite densities were also 37.5% high, 37.5% moderate, and 25% low, while for *P. vivax*, they were 66.7% low and 33.3% moderate. Primigravidae, pregnant women under 20 years’ old and rural inhabitants were shown to have high parasite densities. These younger pregnant women could not have fully established partial immunity to *Plasmodium* species [35]. Similarly, Women in their first pregnancy are more likely to harbor high parasite densities because they lack anti-adhesion antibodies against chondroitin sulphate A (CSA) binding especially *P. falciparum* parasites antigen which develop only after successive pregnancies [35, 36]. Additionally, because primigravidae and pregnant women under the age of 20 are often younger than secugravidae and multigravidae, it is possible that they have weaker partial immunity to *Plasmodium* species [37]. In locations where malaria is endemic, a person’s level of partial immunity to *Plasmodium* species is largely acquired as a result of the number of prior infections and their age [37].

Accurate diagnosis is crucial for developing the best treatment plan as well as for implementing successful malaria control measures and elimination programmes. Misidentification could also have a negative impact on public health by causing improper therapies, which could result in medication resistance and even recurrence [38].

This study showed possible deficiencies of RDT and microscopy-based diagnosis of asymptomatic and low parasite density infections. When compared to RT-PCR, it was identified that RDT and microscopy had false negative rates of 50% and 40%, respectively. These false negative findings may have significant effects on mortality, transmission, and health. Therefore, reliable laboratory diagnosis techniques are the cornerstone of effective malaria control and elimination strategies as well as preventing antimalarial drug resistance. Because of scarce laboratory diagnostic resources, microscopy continues to be the gold standard for identifying *Plasmodium* species [39]. This study, however, demonstrated how RT-PCR techniques have provided outstanding sensitivity.

The most used test for confirming malaria before therapy in sub-Saharan Africa is an HRP2 based RDT. But numerous studies have found that deletion of the pfhrp2 gene significantly reduced the sensitivity of HRP2 based RDTs. False-negative results for HRP2 RDTs could come from parasites missing pfhrp2/3 gene, due to a prozone effect because of antigen saturation caused by high parasitemia, due to low parasitemia [39], either the quality of the RDT manufacture or the high specificity of commercially produced proprietary monoclonal antibodies used on the test devices [40], high concentrations of patient antibodies against HRP2/3 antigens may also contribute to the inhibition of RDT recognition of antigens [41, 42]. A perfect confirming test for malaria diagnosis and surveillance, RT-PCR enables the detection of low-density infections and, more significantly, mixed infections that are overlooked by RDT and microscopy [31]. Real time-PCR is still expensive and not very useful for routine diagnosis, despite being the best diagnostic tool with high sensitivity and specificity.

Generally, even if our study had some limitations such as data collection time was outside the main malaria transmission season and it was health facility based cross-sectional study, our findings indicated the advantages and drawbacks of routine diagnostic tests like other few similar findings done in study areas of African and other continents, and the impacts of APIs in malaria control and elimination program.

### Limitations of the study

Data were collected outside of the main malaria transmission seasons, during the dry season and it was health facility based cross-sectional study.

## Conclusion

This study revealed that prevalence of APIs among pregnant women is high 18.7% by RT-PCR and 11.4% by microscopy but somewhat lower 9.6% by RDT. When we compared RDT and microscopy with RT-PCR they showed less sensitivity 50% and 60% and specificity 99.3% and 100%, respectively. Parasite densities were ranged from 360 to 5120 parasites/µl of blood. The mean parasite density was 3213 parasites/µl for *P falciparum* and 1140 parasites/µl of blood for *P. vivax*.

## Data Availability

All materials and data are available at corresponding author.

## References

[1] WHO. Factsheet on the World Malaria Report. 2022 https://www.who.int/news-room/fact-sheets/detail/malaria [Accessed 25 Dec. 2023].

[2] WHO. Global Malaria Programme: pregnant women and infants 2009.

[3] PMI. President’s malaria initiative Ethiopia 2018, Malaria Operational Plan FY 2018.

[4] Agan TU, Ekabua JE, Iklaki CU, Oyo–Ita A, Ibanga I (2010). Prevalence of asymptomatic malaria parasitaemia. Asian Pac J Trop Med.

[5] Jesse Uneke C (2012). Malaria during pregnancy: incidence, manifestations, therapy, and prevention. Curr Women’s Health Reviews.

[6] Ricke CH, Staalsoe T, Koram K, Akanmori BD, Riley EM, Theander TG, Hviid L (2000). Plasma antibodies from malaria-exposed pregnant women recognize variant surface antigens on Plasmodium falciparum-infected erythrocytes in a parity-dependent manner and block parasite adhesion to chondroitin sulfate A. J Immunol.

[7] Mockenhaupt FP, Ulmen U, von Gaertner C, Bedu-Addo G, Bienzle U (2002). Diagnosis of placental malaria. J Clin Microbiol.

[8] Mayengue PI, Rieth H, Khattab A, Issifou S, Kremsner PG, Klinkert MQ, Ntoumi F (2004). Submicroscopic Plasmodium falciparum infections and multiplicity of infection in matched peripheral, placental and umbilical cord blood samples from Gabonese women. Tropical Med Int Health.

[9] Mockenhaupt FP, Bedu-Addo G, Junge C, Hommerich L, Eggelte TA, Bienzle U (2007). Markers of sulfadoxine-pyrimethamine-resistant Plasmodium falciparum in placenta and circulation of pregnant women. Antimicrob Agents Chemother.

[10] Ordi J, Menendez C, Ismail MR, Ventura PJ, Palacín A, Kahigwa E, Ferrer B, Cardesa A, Alonso PL (2001). Placental malaria is associated with cell-mediated inflammatory responses with selective absence of natural killer cells. J Infect Dis.

[11] Nosten F, Rogerson SJ, Beeson JG, McGready R, Mutabingwa TK, Brabin B (2004). Malaria in pregnancy and the endemicity spectrum: what can we learn?. Trends Parasitol.

[12] Douamba Z, Bisseye C, Djigma FW, Compaoré TR, Bazie VJ, Pietra V, Nikiema JB, Simpore J. Asymptomatic malaria correlates with anaemia in pregnant women at Ouagadougou, Burkina Faso. Biomed Res Int. 2012; 11;2012. 10.1155/2012/198317 PMC3511849 23226937

[13] Menendez C, Ordi J, Ismail MR, Ventura PJ, Aponte JJ, Kahigwa E, Font F, Alonso PL (2000). The impact of placental malaria on gestational age and birth weight. J Infect Dis.

[14] McMorrow ML, Aidoo M, Kachur SP (2011). Malaria rapid diagnostic tests in elimination settings—can they find the last parasite?. Clin Microbiol Infect.

[15] Kahama-Maro J, D’Acremont V, Mtasiwa D, Genton B, Lengeler C (2011). Low quality of routine microscopy for malaria at different levels of the health system in Dar es Salaam. Malar J.

[16] Koita OA, Doumbo OK, Ouattara A, Tall LK, Konaré A, Diakité M, Diallo M, Sagara I, Masinde GL, Doumbo SN, Dolo A (2012). False-negative rapid diagnostic tests for malaria and deletion of the histidine-rich repeat region of the hrp2 gene. Am J Trop Med Hyg.

[17] Gamboa D, Ho MF, Bendezu J, Torres K, Chiodini PL, Barnwell JW, Incardona S, Perkins M, Bell D, McCarthy J, Cheng Q (2010). A large proportion of P. Falciparum isolates in the Amazon region of Peru lack pfhrp2 and pfhrp3: implications for malaria rapid diagnostic tests. PLoS ONE.

[18] WHO. Universal access to malaria diagnostic testingan operational manual. Geneva: World Health Organization. 2013. http://www.who.int/malaria/publications/atoz/9789241502092/en/. Accessed 24 July 2019.

[19] Erdman LK, Kain KC (2008). Molecular diagnostic and surveillance tools for global malaria control. Travel Med Infect Dis.

[20] Mal E (2011). Diagnostics. A research agenda for malaria eradication: diagnoses and diagnostics. PLoS Med.

[21] FMoH F (2012). National malaria guidelines. Malar Diagnosis Treat.

[22] Federal Democratic Republic of Ethiopia. Population census commission: Summary and statistical report of the 2007 population and housing census: Population size by age and sex. Addis Ababa.

[23] Shimelis D, Habtamu G, Getachew A (2011). A cross-sectional study on bovine trypanosomosis in Jawi district of Amhara Region, northwest Ethiopia. Ethiop Veterinary J.

[24] Dinko B, Ayivor-Djanie R, Abugri J, Agboli E, Kye-Duodu G, Tagboto S, Tampuori J, Adzaku F, Binka FN, Awandare GA (2016). Comparison of malaria diagnostic methods in four hospitals in the Volta region of Ghana. Malar World J.

[25] https://www.shoklo-unit.com/sites/default/files/resources/transcript_how_to_perform_a_proper_thick_and_thin_smear_for_malaria_diagnosis.pdf.

[26] Sastry AS, Bhat S. Essentials of medical parasitology. Jaypee Brothers, Medical Publishers Pvt. Limited; 2018;12:60.

[27] Trape JF (1985). Rapid evaluation of malaria parasite density and standardization of thick smear examination for epidemiological investigations. Trans R Soc Trop Med Hyg.

[28] Allen SJ, Bennett S, Riley EM, Rowe PA, Jakobsen PH, O’Donnell A, Greenwood BM (1992). Morbidity from malaria and immune responses to defined Plasmodium falciparum antigens in children with sickle cell trait in the Gambia. Trans R Soc Trop Med Hyg.

[29] Lucchi NW, Narayanan J, Karell MA, Xayavong M, Kariuki S, DaSilva AJ, Hill V, Udhayakumar V (2013). Molecular diagnosis of malaria by photo-induced electron transfer fluorogenic primers: PET-PCR. PLoS ONE.

[30] Harris I, Sharrock WW, Bain LM, Gray KA, Bobogare A, Boaz L, Lilley K, Krause D, Vallely A, Johnson ML, Gatton ML (2010). A large proportion of asymptomatic Plasmodium infections with low and sub-microscopic parasite densities in the low transmission setting of Temotu Province, Solomon Islands: challenges for malaria diagnostics in an elimination setting. Malar J.

[31] Britton S, Cheng Q, McCarthy JS (2016). Novel molecular diagnostic tools for malaria elimination: a review of options from the point of view of high-throughput and applicability in resource limited settings. Malar J.

[32] Wu L, van den Hoogen LL, Slater H, Walker PG, Ghani AC, Drakeley CJ, Okell LC (2015). Comparison of diagnostics for the detection of asymptomatic Plasmodium falciparum infections to inform control and elimination strategies. Nature.

[33] Michelle L, Gatton S, Ciketic JWB, Cheng Q, Chiodini PL, Icardona S, Bell D, Cunningham J (2018). Gozaleze. An assessment of false positive rates for malaria rapid diagnostic tests caused by non Plasmodium infectious agents and immunological factors. PLoS ONE.

[34] Nega D, Dana D, Tefera T, Eshetu T (2015). Prevalence and predictors of asymptomatic malaria parasitemia among pregnant women in the rural surroundings of Arbaminch Town, South Ethiopia. PLoS ONE.

[35] Fried M, Nosten F, Brockman A, Brabin BJ, Duffy PE (1998). Maternal antibodies block malaria. Nature.

[36] Kayentao K, Garner P, van Eijk AM, Naidoo I, Roper C, Mulokozi A, MacArthur JR, Luntamo M, Ashorn P, Doumbo OK, ter Kuile FO (2013). Intermittent preventive therapy for malaria during pregnancy using 2 vs 3 or more doses of sulfadoxine-pyrimethamine and risk of low birth weight in Africa: systematic review and meta-analysis. JAMA.

[37] Filipe JA, Riley EM, Drakeley CJ, Sutherland CJ, Ghani AC (2007). Determination of the processes driving the acquisition of immunity to malaria using a mathematical transmission model. PLoS Comput Biol.

[38] Kang JM, Cho PY, Moe M, Lee J, Jun H, Lee HW, Ahn SK, Im Kim T, Pak JH, Myint MK, Lin K (2017). Comparison of the diagnostic performance of microscopic examination with nested polymerase chain reaction for optimum malaria diagnosis in Upper Myanmar. Malar J.

[39] Bourgeois N, Boutet A, Bousquet PJ, Basset D, Douard-Enault C, Charachon S, Lachaud L (2010). Comparison of three real-time PCR methods with blood smears and rapid diagnostic test in Plasmodium sp. infection. Clin Microbiol Infect.

[40] Nolder D, Stewart L, Tucker J, Ibrahim A, Gray A, Corrah T, Gallagher C, John L, O’Brien E, Aggarwal D, Benavente ED (2021). Failure of rapid diagnostic tests in Plasmodium Falciparum malaria cases among travelers to the UK and Ireland: identification and characterisation of the parasites. Int J Infect Dis.

[41] Ho MF, Baker J, Lee N, Luchavez J, Ariey F, Nhem S, Oyibo W, Bell D, González I, Chiodini P, Gatton ML (2014). Circulating antibodies against Plasmodium Falciparum histidine-rich proteins 2 interfere with antigen detection by rapid diagnostic tests. Malar J.

[42] Kozycki CT, Umulisa N, Rulisa S, Mwikarago EI, Musabyimana JP, Habimana JP, Karema C, Krogstad DJ (2017). False-negative malaria rapid diagnostic tests in Rwanda: impact of Plasmodium Falciparum isolates lacking hrp2 and declining malaria transmission. Malar J.

